# Hospital Meetings

**Published:** 1905-06-17

**Authors:** 


### HOSPITAL MEETINGS.

### CITY OF LONDON HOSPITAL FOR DISEASES OF THE CHEST.

The Governors of the City of London Hospital for Diseases of the Chest held their annual general meeting at Gresham College on the 7th inst. Sir Edward Sassoon, M.P., treasurer of the hospital, took the chair, and in moving the adoption of the report, expressed the profound regret of the Committee at the death of H.R.H. the Duke of Cambridge, who, as far back as 1858 had been the valued patron and friend of the hospital. They had also experienced another heavy loss in the death of Mr. F. D. Mocatta, their vice-president, whose connection with them was as close as his philanthropy was deservedly celebrated. Worthy of mention as proving the hospital's popularity was the fact that there was an increase on the previous year of 6,000 attendances in the out-patients' department. Both donations and subscriptions have shown a slight increase and King Edward's Hospital Fund has given substantial assistance. The new Nurses' Home is now nearing completion and will be honoured at its opening ceremony by the presence of H.R.H. the Duke of Connaught. There is, unfortunately, a debt of about £3,000 still on the building, and for the purpose of liquidating this and also to provide funds for the re-opening of 24 beds, which the hospital has been unfortunately forced to close, an earnest appeal is made for financial assistance. The Chairman paid a warm tribute of thanks for the excellent services rendered to the hospital by Mr. Godfrey Mellor, hon. solicitor, who kindly gives his time and attention gratis, and also by Mr. George H. Masterman, the chairman of the house committee.

The Medical Committee have advised the appointment on the staff of the hospital of a surgeon-dentist. A few changes have also been made in the authoritative power enjoyed by the resident medical officer. For instance, before the suspension of female attendants the cause must now be laid before the committee.

The resolution was seconded and unanimously carried.

Dr. Heron then proposed two resolutions: That Sir Edward Sassoon be re-elected treasurer; and that the appointments as consulting physicians of Sir William Church and Dr. Vincent Harris be confirmed.

Mr. Phibbs and Mr. R. Langton Cole seconded, and the motions were adopted.

The Chairman cordially moved a vote of thanks for the valuable services of the medical staff, and Mr. Hyland, seconding, said he only wished he could express half what he felt for the devotion which they had shown. Dr. Heron replied and said that they (the staff) well knew that the benefits of the hospital were exclusively conferred upon the deserving poor. He strongly advocated the appointment of a dentist.

A vote of thanks to the chairman having been unanimously carried, Sir Edward Sassoon responded by disclaiming the necessity of its being offered on account of the real and sincere pleasure which he felt in assisting a charity so deserving as that of the City of London Hospital.

